# ‘It Was a Bit of a Now or Never Situation’: Experiences of Preconception Care and Support for Women With Multiple Long‐Term Health Conditions

**DOI:** 10.1111/hex.70583

**Published:** 2026-02-18

**Authors:** Stephanie J. Hanley, Sharon McCann, Siang Ing Lee, Danielle Schoenaker, Megha Singh, Ngawai Moss, Nishshanka Mudiyanselage Chamendi Lakshrieni Nishshanka, Zoe Vowles, Rachel Plachcinski, Krishnarajah Nirantharakumar, Mairead Black, Louise Locock, Beck Taylor

**Affiliations:** ^1^ Department of Applied Health Sciences, College of Medicine and Health University of Birmingham Birmingham UK; ^2^ Health Services Research Unit, Health Sciences Building, Foresterhill University of Aberdeen Aberdeen UK; ^3^ School of Human Development and Health, Faculty of Medicine University of Southampton Southampton UK; ^4^ MRC Lifecourse Epidemiology Centre University of Southampton Southampton UK; ^5^ NIHR Southampton Biomedical Research Centre, University of Southampton and University Hospital Southampton NHS Foundation Trust Southampton UK; ^6^ Guy's and St. Thomas' NHS Foundation Trust London UK; ^7^ School of Life Course and Population Sciences, King's College London London UK; ^8^ School of Medicine, Medical Science and Nutrition, Aberdeen Centre for Women's Health Research University of Aberdeen Aberdeen UK; ^9^ Warwick Applied Health, Warwick Medical School University of Warwick Warwick UK

**Keywords:** integrated health care systems, multimorbidity, multiple long‐term conditions, obstetrics, patient centred care, preconception, qualitative research

## Abstract

**Introduction:**

One in five women enters pregnancy with multiple long‐term health conditions, which is associated with increased risks of adverse maternal and child outcomes. There is a lack of research exploring individuals' experiences of preconception care for these women, which is also reflected in existing guidelines that predominantly focus on single health conditions. This study aimed to explore experiences of preconception care and support among women with multiple long‐term health conditions and health professionals.

**Methods:**

This is a secondary analysis of qualitative data collected by the MuM‐PreDiCT consortium. The primary study involved semi‐structured interviews between March 2022 and May 2023 with pregnant (> 28 weeks) and postnatal (< 2 years) women with multiple long‐term physical and/or mental health conditions in the United Kingdom, and healthcare professionals involved in their care. Data captured within the preconception coding reports were analysed thematically.

**Results:**

Fifty‐seven women and 51 healthcare professionals were interviewed. Six themes were identified from the thematic analysis. Women and professionals described the importance of tailored preconception care and support, incorporating condition‐focused counselling (sub‐theme 1) and medication planning (sub‐theme 2). Sensitive and realistic care and support were considered essential, but women had mixed experiences of involvement and empathy from different professionals. The significance of optimising antenatal care by making every preconception contact count was emphasised by both women and professionals, who valued early referrals, specialist input and integration of services. Although professionals viewed the preconception period as an opportunity to empower women, many women felt they had to self‐advocate and seek information due to gaps in professional awareness, knowledge and education. Professionals reported differing views on who, within the care team, should take responsibility for care delivery. Some believed that women should play an active role in managing their health, including initiating conversations around pregnancy intentions. The delivery of preconception care was complicated by a range of challenges, including a lack of service integration, availability, time and funding.

**Conclusion:**

Women with long‐term health conditions can experience substantial gaps in preconception care, characterised by inconsistent guidance and limited access to tailored, reliable support, which frequently leads to feelings of isolation and the need to seek additional information when preparing for pregnancy. These results will inform the co‐development of a care bundle for affected women.

**Patient or Public Contribution:**

Our Patient and Public Involvement group was involved in the design of the study and the analysis and interpretation of the data, and two public study investigators are part of the author group.

## 1 Introduction

One in five pregnant women in the United Kingdom has multiple (two or more) long‐term conditions (MLTCs) before conception [1]. This can include physical and/or mental health conditions, such as epilepsy, diabetes, depression and schizophrenia. MLTCs are associated with adverse maternal, neonatal, and child health outcomes, such as maternal morbidity or mortality, low birth weight and preterm birth [2, 3, 4, 5]. This work, therefore, focused on women with MLTC in order to capture the interacting and cumulative effects of multiple conditions on preconception care, which are not accounted for within single‐condition frameworks or guidelines.

The World Health Organization defines preconception care as ‘the provision of biomedical, behavioural and social health interventions to women and couples before conception’ with the aim of improving health status and reducing behaviours, individual and environmental factors that could contribute to poor maternal and child health outcomes [6]. For the general population, universal preconception care includes folic acid supplementation and supporting those with modifiable lifestyle risk factors (e.g., smoking, obesity, alcohol). For women with MLTC, targeted preconception care would additionally include optimising the management of their conditions and reviewing their medications [7].

Although evidence specific to women with multimorbidity is limited, studies in women with *single* long‐term conditions suggest that engagement with preconception care can evoke emotional distress, particularly due to concerns about potential pregnancy complications [8]. Nevertheless, these studies consistently demonstrate that preconception care is beneficial, with improvements in key pregnancy outcomes, including reduced risks of small for gestational age infants, low birth weight, preterm birth, congenital anomalies, and miscarriage [9]. In some settings, there is preconception guidance for some single long‐term conditions, which provide structured medication review and disease‐specific counselling [9]. However, these models are designed around one condition at a time and do not address the combined or interacting needs of women with MLTCs. Even where such single‐condition pathways exist, implementation in routine practice is inconsistent, and many eligible women do not receive structured preconception support [10, 11].

Two recent qualitative studies explored the pregnancy experience of women with MLTC [12, 13]. Hansen et al. found that women in Denmark with single conditions or MLTC experienced feelings of reservation at the start of pregnancy, and pressure to plan their pregnancies in consultation with healthcare professionals [13]. Hanley et al. identified the importance of medication management in preconception care for UK women with MLTC. Both studies described a lack of comprehensive postnatal care, though limited findings were presented relating to preconception and interpregnancy care [12].

The lack of literature exploring preconception care in women with MLTC is reflected in preconception care guidelines, which focus on single health conditions [10, 11, 14]. However, women contemplating pregnancy with MLTC face unique challenges, including a need for coordination between clinical specialities. Women are more likely to take multiple medications with potential overlapping foetal risks and substantial implications for preconception care [12, 15]. Significant barriers exist in the delivery of preconception care, particularly for individuals with complex health needs, as health professionals often face constraints related to time, funding, and knowledge gaps [16].

This study aims to explore experiences of preconception care and support among women with MLTC and health professionals.

## 2 Materials and Methods

### 2.1 Study Design

This is a secondary analysis of qualitative interview data collected by the MuM‐PreDiCT consortium. Drawing on Heaton's five categories of secondary analysis, this paper is primarily a supplementary analysis, involving a more detailed examination of a specific aspect of the data partially addressed in the primary study [17]. It incorporates elements of supra‐analysis through exploration of new theoretical, empirical or methodological questions that extend beyond the original aims. Lead author (S. J. H.) coordinated the study and led data collection and analysis.

The study adopted an interpretivist approach to understand experiences of maternity care for women in the United Kingdom with MLTC, from the perspectives of service users (*n* = 57) and providers (*n* = 51). To support the interpretation of findings, we summarise key sample characteristics from the primary study here. Women represented all four UK nations, ranged from 18 to over 40 years, and reflected diverse ethnic backgrounds, including White, South Asian, Black, Mixed, and Eastern European groups. They reported a wide spectrum of conditions—58 physical and 15 mental health or neurodevelopmental conditions, with most living with two or more, and many experiencing both physical and mental health comorbidities. The 51 healthcare professionals were similarly diverse, working across 24 clinical roles spanning community and hospital midwifery, obstetrics, maternal–foetal medicine, anaesthetics, psychiatry, neurology, haematology, diabetes, neonatology, general practice and public health (details in Table 1). Full methodological detail and comprehensive participant characteristics are available in the primary study [12].

**Table 1:** Characteristics of participants included.

Women with MLTC (*N* = 57)	
Country of residence	England 37, Scotland 12, Northern Ireland 4, Wales 4
Urban/rural residence	Urban 43, Rural 14
Age (years)	18–25: 5; 26–30: 13; 31–35: 22; 36–39: 16; ≥ 40: 1
Ethnicity	White British/Scottish/Irish/Other White: 39; Mixed: 3; Pakistani: 3; British Asian: 1; Malaysian: 1; Indian Arab: 1; Black British: 2; Black African: 1; Black Caribbean: 1; Nigerian British: 1; White South African: 1; White Polish: 1; Other White: 1; Romanian: 1
Number of long‐term conditions per woman	2 conditions: 27; 3 conditions: 13; 4 conditions: 9; 5 or more: 8
Condition type combinations	Physical only: 25; Mental/neurodevelopmental only: 3; Both physical + mental/neurodevelopmental: 29
Examples of health condition categories — Physical	Neurological: 14; Rheumatic: 11; Gastrointestinal: 9; Cardiac/cardiovascular: 10; Gynaecological: 6; Endocrine (e.g., diabetes): 6; Respiratory: 6; Obesity: 4; Blood disorders (e.g., VTE): 3; Cancer: 1; Skin disorder: 1; Renal: 1; Deafness: 1; Blindness: 1; Other: 4
Examples of mental health/neurodevelopmental conditions	Anxiety: 18; Depression: 13; PTSD/C‐PTSD: 7; Personality disorder: 3; Psychosis: 1; Bipolar: 1; OCD: 1; Anorexia nervosa: 1; Dyspraxia: 2; ADHD: 2; Autism: 1; Learning disability: 1; Other (e.g., trauma‐related dyslexia): 1
Healthcare professionals (*N* = 51)	
Country of residence	England 39; Scotland 9; Northern Ireland 2; Wales 1
Professional roles — Midwifery	Community midwife: 2; Maternal medicine consultant midwife: 1; Epilepsy‐specialist midwife: 1; Maternal medicine midwife: 1; Infant‐feeding specialist midwife: 1; Perinatal mental‐health midwife: 1; Infectious diseases midwife: 1; High‐risk pregnancies midwife: 1; Public protection midwife: 1; Senior midwife coordinator/research midwife: 1; Hospital midwife: 1
Professional roles — Maternity/obstetric/neonatal physicians	Consultant obstetrician and gynaecologist: 3; Consultant obstetrician: 3; Consultant foetal and maternal medicine: 3; Specialty trainee (obstetrics): 3; Consultant anaesthetist: 2; Consultant neonatologist: 1; Specialty trainee neonatologist: 1; Consultant obstetric physician: 1; Obstetric physician + consultant cardiologist: 1
Other secondary care clinicians	Consultant psychiatrist: 2; Consultant haematologist: 1; Diabetes consultant: 1; Advanced nurse practitioner (epilepsy): 1; Consultant neurologist: 1
Primary care/public health roles	General practitioner: 3; Health‐visitor/infant‐feeding lead: 1; Consultant public health: 1

Secondary in‐depth inductive thematic analysis of relevant coded data was undertaken [18], focusing specifically on data labelled in earlier rounds of coding under ‘preconception counselling’ (interviews with women) and ‘preconception pathways’ (interviews with healthcare professionals).

The study team included individuals with academic and clinical expertise and lived experience of MLTC in pregnancy. Throughout the process, the research team considered their positionality, specifically their disciplinary and clinical backgrounds, personal experiences, and their influence on interpretation.

### 2.2 Data Collection

Data collection for the primary study was completed between March 2022 and 2023. Interviews were conducted online (MS Teams or Zoom), by telephone or in‐person in participants' homes by S. J. H. and S. M. Interviews lasted 45–60 min. Reflective notes were recorded throughout. Interview guides were developed with the MuM‐PreDiCT patient and public involvement and engagement (PPIE) group. Women were asked how their health conditions affected their daily lives and their pre‐pregnancy management. Professionals were asked about their role in MLTC care before, during and after pregnancy. Interviews were digitally recorded and transcribed verbatim. NVivo 14 was utilised to support data management and analysis [19].

### 2.3 Data Analysis

Analysis in the primary study was interpretive. Data were analysed inductively using thematic analysis [18]. A coding framework was developed to support the iterative refinement of codes into main themes and sub‐themes.

In this secondary analysis, (S. J. H.) inductively coded the data in the preconception care coding reports from the primary analysis. Twenty‐eight codes were identified from women's interviews, and 40 codes from professional interviews. Following the first round of coding, S. J. H. and S. M. created two ‘one sheet of paper’ (OSOP) summaries to identify patterns in the data in interviews with women and professionals [20]. OSOPs were used in online research team meetings to facilitate discussion and interpretation. Members engaged in further reflection and online discussion to develop codes into potential themes. To support analytical discussions, S. J. H. and S. M. revisited the interview transcripts as needed.

Research team discussions were supported by input from the MuM‐PreDiCT PPIE group. The PPIE group includes women with MLTC and recent experience of maternity care in the four countries of the United Kingdom. Two rounds of PPI engagement directly informed the analysis. A general discussion of the qualitative dataset (March 2023) with nine contributors with lived experience or PPIE representation. The second, a dedicated meeting (May 2025) with eight contributors focusing specifically on preconception care for women with MLTC. In these sessions, anonymised extracts from interviews with women and clinicians were discussed, helping to identify and prioritise key issues, including gaps in medication support, fragmented care, the emotional burden of self‐advocacy, the value of specialist‐led preconception counselling, and the importance of early, accessible information. These discussions directly shaped the interpretation of the interview data and the framing of the analysis. Two PPIE co‐authors also contributed throughout, including proposing the focus on preconception care, highlighting priority areas, and helping translate findings into practical principles for care. This dual approach ensured that both group‐level feedback and focused PPIE contributions informed the development and finalisation of the manuscript.

All members of the research team and PPIE contributed to theme development and agreed on the final results. Extracts are labelled W for women and S for staff, alongside the participant number.

## 3 Results

Six main themes were developed: four captured findings from both women and professionals, and two captured professional perspectives only. We start by highlighting the key areas preconception care focuses on (long‐term conditions and medication) and the manner in which it should be delivered for women with MLTCs. We then discuss the optimal time preconception care should occur, highlighting that every contact with a health care professional in the preconception period counts. Finally, the themes discuss the facilitators and barriers to preconception care, including women's and professionals' awareness and knowledge of preconception care, clarity in whose responsibility it is, and health system barriers to delivering preconception care.

### 3.1 Theme 1: Tailored Preconception Care and Support

#### 3.1.1 Subtheme: Condition‐Focused Counselling

Both women and staff described the preconception period as a critical opportunity to optimise women's health before pregnancy. Women valued the support and reassurance provided by specialist teams and described dedicating significant time and effort to work with their specialist to achieve optimal management of their health conditions.

I take morphine on a daily basis, and this was something I discussed again years in advance … I've had pain my whole life because of my Ehlers‐Danlos, essentially the pain team were really amazing and they were just like, “Look, we need you to be as well as possible throughout your pregnancy, so like … So, they were really reassuring, and they were really brilliant about it.”

(W17, Ehlers‐Danlos syndrome, thyroidectomy following thyroid cancer, breast cancer, long‐term chemotherapy fatigue, dyspraxia, dyslexia)

…pre‐pregnancy is where you would try and see women with multiple conditions … one of the most important things can be contraception … because if you're going to take on a high‐risk pregnancy having it when you want it is ideal, and when your medication is sorted, and when you're in tiptop condition as far as your condition can be.

(S15)

However, due to the progressive nature of certain health conditions, some women described being encouraged to plan their pregnancy before their health deteriorated. In these situations, treatment decisions were often closely tied to reproductive planning, leaving women feeling pressured into making time‐sensitive choices.

My rheumatologist was like, “You want children, I think you crack on because at the end of the day even if you have no health problems, we're all just slowly deteriorating, and unfortunately for you that's got other connotations with ank spond and fibromyalgia.”

(W26, Ankylosing spondylitis, fibromyalgia, 5 months postpartum)

It was a bit of a now or never situation, so it was like you either have a baby now and then we start treatment afterwards, or we start treatment now, you have to get used to it, spend a few years on it, then we have to wean you off it for a year, and then find something else that's suitable for pregnancy.

(W15, Non‐epileptic attack disorder, anxiety, depression, 4 months postpartum)

It was a bit of a now or never situation, so it was like you either have a baby now and then we start treatment afterwards, or we start treatment now, you have to get used to it, spend a few years on it, then we have to wean you off it for a year, and then find something else that's suitable for pregnancy.

(W15, Non‐epileptic attack disorder, anxiety, depression, 4 months postpartum)

Both women and staff identified missed opportunities to support lifestyle changes and weight management, despite the potential to reduce the severity of their health conditions and increase the likelihood of natural conception. Gaps in mental health support were also raised, especially among women with previous pregnancy loss.

I think [preconception care] also fails if ladies don't have comorbidities at that point but they do have multiple risk factors, like smoking and BMI … and there's been no input to address them … then when they do become pregnant they then get multiple comorbidities, and then get diabetes and may have pre‐eclampsia all of [a] sudden…

(S33)

I did say, “It's happened again, I'm really struggling with my mental health,” and he [GP] just said that he was going to refer me to gynaecology at that point to see what was happening … so, when I had my third [miscarriage], which was earlier this year in April … I did call the GP again, and I had a big breakdown, and I just said it's too much for me, and that's when he did say, “Okay, maybe you should try some antidepressants.”

(W41, Raised BMI, endometriosis, 32 weeks pregnant)

Professionals described varied availability of preconception counselling, with established pathways for specific conditions, such as diabetes, and limited provision elsewhere. One diabetes consultant attributed this discrepancy to high‐risk care being delivered at specialist centres outside of a woman's local area.

So, our endocrinologists have got it nailed [for diabetes], our neurologists, there's not a process in place, our cardiologists, there's not a process in place, partly because a lot of the high‐risk things in cardiology will be managed by a different hospital, than local to the patient.

(S20)

A holistic approach to risk counselling was seen as essential, addressing how pregnancy may affect existing conditions, risks to the baby, and the potential for pregnancy‐related complications. One consultant in foetal and maternal medicine emphasised the often overlooked need to support women's mental health from preconception and throughout pregnancy.

We very much say, “Okay well these are the risks to the baby or to the pregnancy of your heart condition, and these are the risks to the heart condition of the pregnancy, and this is how we can … this would be the best way to approach it, but once you become pregnant we'll totally support you, we'll look after you,” … you probably need to additionally address the mental health aspect of that. So, we probably need to be saying, “It will be a huge challenge to your mental health this whole journey,” and offering perinatal mental review or a specialist mental health review … and I think that is something that's totally overlooked.

(S07)

#### 3.1.2 Subtheme: Medication Planning

Women and professionals described the preconception period as critical for medication planning, including dose adjustments and transitioning to pregnancy‐compatible treatments, while balancing the risk of exacerbation of previously well‐controlled health conditions and potential teratogenic effects.

I was on Methotrexate, they'd said to me, “If you get pregnant on this, it's really serious,” I think it causes deformities or something … they were basically like, “You'd have to get an abortion. So, it was obviously really important to me that I was on drugs that would be okay to try for a baby on, but it took my … I have ulcerative colitis and it … my condition took a very long time to get under control, so it was quite a long process just coming off that drug and finding a drug that worked.”

(W21, Ehlers‐Danlos Syndrome type 3, ulcerative colitis, postural orthostatic tachycardia syndrome)

Professionals highlighted the importance of coordinated multidisciplinary medication management, in partnership with women. This was considered essential for reassuring women about medication safety and supporting informed decision‐making regarding maternal and neonatal risks. A multidisciplinary approach was seen as particularly vital in complex cases, such as those involving severe mental health considerations.

Getting them on pregnancy safe medication, and just talking through what will pregnancy look like, and for all of the conditions that we do it's all about what will happen to the disease with pregnancy, what could pregnancy do to their disease, what could the disease do to their pregnancy, and women understandably are often worried about medication. So, it's reassuring them that we need to keep their disease as quiescent as possible, and that there are actually a lot of safe medications we can give them that are much safer than stopping it and then talking to them about what might happen.

(S17, Consultant obstetrician)

Although many professionals recognised the value of early pre‐conception medication consultations, they often did not occur. As a result, necessary changes were instead made during pregnancy against time pressures.

It actually takes a long time of precious time during pregnancy that would be good if we met them pre‐pregnancy, because we can talk about what would be the plan if you are pregnant, and this is what we would suggest, and women can have time to digest it and consider it, rather than we see them at eight weeks/ten weeks pregnant, and they've stopped their medications, and the clock is ticking.

(S45, Consultant cardiologist and obstetric physician)

### 3.2 Theme 2: Sensitive and Realistic Care and Support

Women reported varied experiences of preconception support and involvement, with some describing more comprehensive care before their first pregnancy compared to subsequent pregnancies. Some women described feeling well‐supported by their GP, citing timely referrals and appropriate guidance as key factors reassuring them that pregnancy was a possibility. For example, one woman reported feeling reassured by the offer of enhanced perinatal mental health support.

I think it gave me some confidence that women who have bipolar and anxiety can have successful pregnancies, and they made me really hopeful that I wouldn't have an episode if I followed everything as much as I could and got as much support as I could.

(W14, Bipolar, anxiety, 16 months postpartum)

However, other women received little to no input from specialist teams and described varied input from obstetric teams. For example, while one woman described a preconception consultation with an obstetrician to discuss ‘the effect of IVF treatment and me getting pregnant with all my medical conditions’ (W46, Cardiomyopathy, epilepsy, PCOS, endometriosis, anxiety, depression, 11 months postpartum), another was declined a preconception obstetric consultation.

He [GP] made the referral for me and then Obstetrics declined the referral and said there was nothing they could do to help me until I got a positive test, which, to me, is just bonkers because as soon as you've got that positive test, I have a period of about two weeks where I'm well before it hits … it would have been so much better and that was just … I tried but it was just declined.

(W27, Hypothyroidism, hypogonadotropic hypogonadism, anxiety, depression, 11 weeks postpartum)

Regarding the delivery of preconception counselling, some women described a lack of empathy from healthcare professionals, as in the example below.

It was not nice the whole thing, I was not supported at all. My support was my husband, and he was going through it as well. It was, the medical professionals were very matter of fact, no empathy at all. It was like well do it or don't, we're giving you the option sort of thing. I didn't know, I was like what you're throwing this diagnosis at me, telling me to make a decision that is going to change my life, and could make me more poorly.

(W16, Non‐epileptic attack disorder, anxiety, depression, 4 months postpartum)

In contrast, professionals emphasised the importance of facilitating shared decision‐making with the woman and her family to support the delivery of sensitive and realistic preconception care. Key enablers to shared decision‐making included the provision of comprehensive, individualised advice; the use of evidence‐based resources to guide discussions; and enhancing women's understanding of their own health conditions and the potential interactions with pregnancy.

Prenatal counselling point of view, I think I feel relatively confident discussing things with the use of websites, we've got resources like the Medicines in Pregnancy, because it's all just very clear, and it's got the scientific background for those recommendations … I use that with the patient, and I'm really keen on shared decision‐making … weighing up the information that we've got and letting them make an informed decision whilst also gently pushing the stats and figures where necessarily just to help them with that.

(S40, General practitioner)

Additionally, a few professionals highlighted the importance of the setting in which preconception counselling is delivered, emphasising the need for sensitivity and, in some cases, the avoidance of maternity settings, especially for women with complex health issues, where conversations may be difficult.

Because at the [hospital name]…, so you might see women wandering round with babies already, so not only the pregnancy bumps…. But so, I think the setting for that is quite important that ideally you would make it in a non‐maternity setting, but with maternity professionals there so that then you can give the information that the women need and families need.

(S24)

### 3.3 Theme 3: Optimising Antenatal Care: Making Every Preconception Contact Count

The importance of preconception counselling in facilitating timely and tailored antenatal care was evident across professionals' and women's accounts. Some women reported receiving comprehensive preconception guidance, including structured early pregnancy care plans, often including expedited referrals and early engagement with specialist teams.

She told me that once I was pregnant not to go through my GP, to make it all a slow process, to contact them directly to say I'm pregnant, and they'll get me straight in on the obstetric systems for certain clinics, for the heart clinic specifically, but with epilepsy support.

(W46, Cardiomyopathy, epilepsy, PCOS, endometriosis, anxiety, depression, 11 months postpartum)

Professionals highlighted that complex antenatal care planning was most effective when women were well‐informed and actively engaged, often due to prior counselling.

Women that need a lot of antenatal care in my experience they're used to being quite medicalised, they're used to a lot of hospital appointments … they know because of their physicians that when they, or at least they should have been told that actually when you're thinking about starting a family get into contact, we want to optimise your condition before you become pregnant to ease the pregnancy.

(S05)

Staff also described that early pre‐pregnancy engagement could optimise antenatal care and improve outcomes, while unmanaged conditions and unplanned pregnancies increased risks and complexity during pregnancy.

What you want is to be able to have that preconceptual chat. So ideally you don't want unplanned pregnancies in women who are going to need a lot of care during their pregnancy time, and I feel like that is probably if I have to say the one thing, I think makes a best … the biggest difference, is that.

(S11, Consultant in obstetrics and gynaecology)

There was strong support among staff for embedding reproductive health discussions throughout the care pathway, starting from adolescence and continuing through adult services. One epilepsy specialist midwife noted the value of early engagement through paediatric to adult transition clinics by saying that ‘having that opportunity of meeting young people aged 15 or 16 and then being part of their pathway through is optimal’ (S03).

Despite this, staff acknowledged that conversations around family planning were inconsistently raised, particularly during treatment decisions. One diabetes consultant highlighted gaps in specialist reviews for women with type 2 diabetes by saying:

We need to make sure … it becomes part of their annual review … a discussion around contraception, and that discussion around if there are significant problems related to their diabetes or other health conditions, why they would need to think about pregnancy and planning for it, rather than just becoming pregnant.

(S29, Diabetes consultant)

Others called for broader public health strategies, including school‐based education, to normalise preconception counselling.

There's a lot around not getting pregnant in schools, which is absolutely fine, but actually it almost needs to start there to say actually preparing for pregnancy is really important whether you've got a medical problem or not, but particularly if you've got a medical problem, and making sure people perhaps then remember where they can access care.

(S17, Consultant obstetrician)

### 3.4 Theme 4: Awareness, Knowledge and Education

Women often described feeling a strong personal responsibility to seek information, manage medication risks and mitigate against potential complications, largely due to inconsistent or insufficient guidance from professionals. Many women reported the need to self‐advocate, seek second opinions, or rely on peer support and online resources to inform decisions.

I'd done so much research and, and had so much support from Pregnancy Sickness Support before I got pregnant … I was able to give the names of all these drugs … I'm on a drug at the moment called Xonvea, which no GP has even heard of.

(W27, Hypothyroidism, hypogonadotropic hypogonadism, anxiety, depression, 11 weeks postpartum)

Some women felt reassured during preconception counselling but later experienced a gap between what had been promised and the care received during pregnancy.

They really sounded like they dealt with this all the time, and it wasn't a problem, and they knew how to handle it, and then when I actually got pregnant and managed to stay pregnant, they really didn't know how to handle it.

(W16, Ehlers‐Danlos syndrome, spondylolisthesis, severe asthma, functional neurological disorder, PTSD, 11 months postpartum)

Continuity of care and established relationships were also seen as essential for initiating sensitive conversations around pregnancy, as, without these, staff found it harder to raise complex topics.

It's not something I would normally do off my own back if it was the first time I met the patient. I think you need to make sure that that's not going to be misunderstood or go down badly, but equally I think it is really important that these patients are aware of issues around conception and pregnancy.

(S40, General practitioner)

However, staff acknowledged barriers to engagement, particularly for women in deprived circumstances or with complex lives, making sustained follow‐up more difficult.

The number of women I've seen and said, “You know, you need this treatment,” or, “We need to see you again,” and they never go back to their GP, or they lose the prescription, or they don't have a follow‐up, or move house … chaotic, busy lives, you know particularly with deprivation means that health isn't as much as a priority than if you live in an affluent area and you've spent years thinking about your pregnancy, you've done all the research around it, and you know exactly who to contact.

(S38, Consultant rheumatologist and obstetric physician)

To strengthen support, staff advocated for greater use of evidence‐based resources and closer collaboration between primary care, specialist services, and obstetric teams. In England, the introduction of Maternal Medicine Networks in 2021 was seen as a key mechanism to support coordinated preconception care for women with MLTC [21].

She may say to them [diabetes or kidney consultant], “I am planning for pregnancy, how are things now?” And if the doctor thinks it may not be safe, or it might be safe, whatever, they say, “Okay, this is what the knowledge I have, but I will refer you to the obstetric team who will be able to give you more information regarding that.”

(S15, Maternal medicine consultant)

### 3.5 Theme 5: Responsibility of Preconception Care

Staff reflected that preconception care is often provided only on request, highlighting gaps in shared responsibility across specialities. They emphasised the need for broader education across specialities and clearer roles to ensure reproductive health is addressed proactively.

Preconceptual advice or care is usually on request … it's not just about obstetricians have that role … you're a cardiologist, you have someone who is of reproductive age, they've got a significant cardiac condition, I feel that role of contraception and family planning sits within that. So, it's about that general education and knowledge, and that has to sit with everybody, not just us as obstetricians.

(S11, Consultant in obstetrics and gynaecology)

Staff also highlighted that reproductive planning, especially around contraception and medication planning, is often inconsistently addressed due to unclear professional responsibilities. One consultant rheumatologist and obstetric physician highlighted the importance of specialist teams taking ownership of contraception counselling.

The number of rheumatologists I talk to, I'm like, “Contraception is your business.” They were like, “Why, and it's GPs, it's sexual health doctors.” I'm like, “But you're the one seeing these patients, you're the one that's putting them on treatments that are not compatible with pregnancy … the drug regulators made it very clear, it's the prescriber's responsibility to discussing contraception.”

(S38)

Pre‐pregnancy counselling was perceived to be targeted towards women with severe conditions, while those with mild or moderate disease were often overlooked. Some staff suggested that colleagues lacked confidence or expertise in managing complex cases, particularly where multiple risk factors or contraindicated medications were involved. In such cases, specialist input and shared care responsibility were considered essential.

We tend to see the very high‐risk women that are on medications that are not compatible with pregnancy, and that actively told someone that they want to get pregnant … we have a massive bias for the very, very sick, because that's the ones that our colleagues are not comfortable with, but we should see a lot more, we should see the moderate disease, and sometimes even the mild disease.

(S45, Consultant cardiologist and obstetric physician)

While staff supported shared care responsibility, some believed women should take an active role in their health and initiate discussions around pregnancy intentions. However, staff acknowledged that women's engagement with preconception counselling varies considerably, with some experiencing barriers to engagement, including complex life circumstances, limited understanding, or a lack of awareness about its importance. Staff shared that when women are able to engage with preconception planning, it often leads to improved pregnancy outcomes through tailored care and optimal preparation.

It's a huge spectrum, a huge spectrum of people who are absolutely nailing and on top of it and it's really organised to the extent that they're hyper anxious about it … and there's other people … we've tried to speak to them about pregnancy planning, just doesn't always sink in and they've got other things going on in life or challenges with how they absorb that kind of information.

(S20, Diabetes consultant)

Staff also noted that early, sustained relationships support continuity of care and help women to feel more confident when discussing preconception planning. However, pregnancy intentions are often not disclosed, and staff miss opportunities to initiate conversations. One consultant obstetrician (diabetes lead) suggested that this communication gap may stem from inconsistent messaging, limited understanding among women or a lack of proactive discussion initiated by staff.

I don't know where that gap lies, whether it's early diabetes discussions, or it's when they get diagnosed with type 1/type 2, “Oh by the way if you're ever trying to get pregnant let us know, because we need to make sure you're as fit as you can be, because there can be risks.” Either I don't know, we're not telling them that as often, or it's not registering, or they aren't coming to tell us when they want to get pregnant.

(S36)

### 3.6 Theme 6: Challenges to Preconception Care Delivery

Staff highlighted several systemic and operational barriers to delivering preconception care for women with MLTC. Barriers included limited funding, lack of service integration, and unclear ownership. As one obstetric physician explained:

The cornerstone for women with multiple conditions is pre‐pregnancy counselling: it's a service that is very poorly funded … it's not funded by Obstetrics because these women aren't pregnant, and physicians don't really see it as an important part of their work because they've got so many other demands to meet.

(S38)

Despite such barriers, staff acknowledged the importance of preconception care and noted that referrals still took place, even in the absence of a formal service.

I think preconception care is not so well embedded in the system … very well acknowledged by most of us that it is essential, and we do promote it. We do get the referrals from the consultants, from the other medical specialities if they have explored that.

(S15, Maternal medicine consultant)

Staff also raised concerns related to care access. One consultant obstetric physician noted that, while tertiary centres often had established care pathways, smaller hospitals lacked such provision, resulting in substantial gaps in support for women with complex health needs.

The tertiary centre in which I work, which is [hospital name], and so pre‐pregnancy counselling is a great example there…. but places like [hospital name] have a high … a large amount of complex medicine, and women who really need counselling, but the least access to it.

(S23)

Several staff identified time constraints as a barrier to preconception care. Staff called for protected time and longer appointments to address complex health needs, and women's and family members' concerns.

It's a much more detailed conversation … where all the questions could be addressed and talked through, not just the medical stuff, their general concerns and questions … often they're with someone … and they might have some questions. So, I think it's something that needs to be done in a safe and non‐time pressured environment.

(S22, Consultant intensivist and obstetric physician)

Staff highlighted specific challenges in providing contraception advice and access as part of preconception care, noting that many pregnancies among women with MLTC are unplanned, leading to complex and emotionally difficult decisions about whether to continue with a pregnancy. Staff described ongoing efforts to improve contraception counselling but reported that access has declined in recent years, with many women still encountering barriers to obtaining contraception.

Even before the pandemic, the All‐Party Parliamentary Group who were on reproductive health reported about the limit of access to contraception, how access has fallen in recent years … the number of women we see, they say, “Oh, I just didn't know to access it,” or “I couldn't get through to them.”

(S38, Consultant rheumatologist and obstetric physician)

Some staff proposed solutions to enhance the integration and uptake of preconception care for women with MLTC. For example, one specialist midwife (perinatal mental health) suggested that ‘everyone with a history of anxiety and depression, or a family history of a mental health condition can have at least one [preconception] appointment’ (S34). Suggestions were made to enhance accessibility, including better signposting to counteract slow referral processes and the use of patient‐initiated follow‐up, enabling women to proactively seek preconception advice.

Patient initiated follow‐up, which is essentially when a patient is stable you discharge them … but as part of your re‐referral criteria they can refer themselves back in … it's about the patients knowing that if they were planning a pregnancy, it's very quick and easy for them to get advice.

(S35, Advanced nurse practitioner (epilepsy)

## 4 Discussion

### 4.1 Main Findings

Study findings indicate that the preconception period presents a critical opportunity to optimise the health and medication management of women with two or more pre‐existing health conditions. However, this opportunity is often missed, with care frequently centred around individual health conditions rather than a holistic approach. In the absence of comprehensive, integrated or consistent guidance from healthcare teams, women often conduct their own research when preparing for pregnancy, which leaves them feeling isolated and unsupported when facing complex decisions about starting or expanding their family. While women's and specialists' perspectives often aligned, there were examples suggesting discordance. In particular, some women reported feeling that their requests for preconception support were not acknowledged, while, in contrast, specialists emphasised the value of these discussions and having time to offer them. This may reflect diverse geographical and practice contexts and different professionals' behaviour. Systemic challenges in providing care may also limit specialists' ability to offer optimal support. Professionals participating in this study may also have been more likely to prioritise preconception care, compared with professionals who did not choose to take part. The delivery of preconception care was complicated by a range of challenges, including a lack of service integration, availability, time and funding.

### 4.2 Results in the Context of Existing Literature

In this study, women and healthcare professionals recognised that the preconception period is an important opportunity to optimise long‐term condition and medication management. These components of preconception care are listed in the International Federation of Gynaecology and Obstetrics' (FIGO) preconception checklist for women with long‐term conditions [22]. For these women, preconception care has been shown to improve healthy behaviours, disease control and pregnancy outcomes, and reduce the risk of severe maternal morbidity [9, 23].

Despite its importance, study participants reported that the opportunity for preconception care is often missed. Similar to our study, Hammarberg et al.'s systematic review (2022) reported that women with long‐term conditions have unmet preconception health information needs, resulting in anxiety about their pregnancy [24]. Information that is pertinent to women included medication safety, medication adjustment and pregnancy impacts on long‐term conditions [24, 25].

Previous studies have quoted clinicians' limited time and knowledge as barriers to preconception care [25, 26, 27]. In our study and the existing literature, there is a lack of consensus regarding which healthcare professional(s) is/are responsible for leading or providing preconception care [26, 27]. Admiraal et al. (2022) found that women with inflammatory bowel disease and rheumatological disease preferred a joint preconception consultation with their disease‐specific specialist and an obstetrician [25].

Our study found some expectations for women to initiate preconception conversations with healthcare professionals. However, this can lead to inequality in accessing preconception care. Steel et al.'s systematic review (2015) on the use of preconception services by women with long‐term conditions (mainly diabetes mellitus) reported that a minority, between 18% to 45%, initiated and engaged in preconception care [8]. The literature reports significant gaps in women's knowledge of pregnancy risks associated with long‐term conditions and the availability of preconception care [8]. A review by Zace et al. (2022) showed that women need to be made aware of the importance of seeking preconception medical care and medication review for their long‐term conditions [28]. Studies have shown that awareness of preconception care among women was lower in those with lower education levels and from ethnic minority backgrounds [26]. Nana et al.'s systematic review (2023) found that women with higher education and job security were more likely to receive preconception care [9].

### 4.3 Strengths and Limitations

This secondary analysis drew on a rich, high‐quality dataset from a UK‐wide study, incorporating diverse perspectives from 57 women with various combinations of physical and mental long‐term conditions and 51 healthcare professionals across different services. The original study was methodologically robust and reported in detail, enhancing the credibility of this secondary analysis. The multidisciplinary research team, with academic, clinical, and lived experience expertise, brought valuable reflexivity and depth to the interpretation. The use of inductive thematic analysis allowed themes to be developed organically. Working with our PPIE group ensured the findings were relevant and grounded in lived experience. Moreover, this analysis offered an efficient and ethical way to maximise the use of existing data, particularly valuable when researching sensitive subjects.

It is important to note that in this secondary analysis, the original interviews were not specifically designed to explore preconception care in‐depth, which may limit insights into certain areas, such as women's preferred providers for preconception health discussions. Analysis was restricted to pre‐coded data categories, potentially overlooking uncoded but relevant content. In addition, there was no opportunity for participant validation, which may slightly limit the ability to confirm the interpretation of participants' accounts. Involvement of some researchers in both the primary and secondary studies may have influenced interpretation. While the sample was diverse with respect to health conditions, ethnicity and geography, the study did not recruit women who required an interpreter despite extensive efforts to do so, limiting representation of linguistically marginalised groups.

### 4.4 Clinical and Research Implications

Women with MLTC require a multidisciplinary and woman‐centred approach to pregnancy planning and preparation (Box 1). While preconception care is currently relatively well‐coordinated for women with diabetes and epilepsy in the United Kingdom, this is generally less consistent for those with other individual or multiple health conditions. Shared responsibilities and effective communication across primary care, specialist teams, maternal medicine teams, and women are important to provide tailored, condition‐specific guidance and MLTC care. This includes ensuring continuity of care during the transition to adult services for women diagnosed with MLTC in childhood, integrating pregnancy intention conversations into routine clinical encounters and medication reviews, and offering ongoing support before, during, between and after pregnancy. Enhanced training for health professionals and clear care pathways are needed for high‐quality and woman‐centred support. Our findings suggest that optimised care pathways should include health behaviour and weight management interventions, mental health care, and education. Prioritising and adequately funding preconception care service integration will be essential to achieving this.

Box 1: **Core components of good preconception care for women with MLTC.**

This table summarises the core elements of good preconception care for women with multiple long‐term conditions drawn from qualitative findings with women and health professionals.

**Table:** 

Theme	Key component	Good practice principles	Example
Core components of good preconception care			
Tailored care (Condition‐focused counselling)	Clarifying risks of pregnancy	– Honest and clear explanation of the benefits and risks to the woman and foetus – Discussing changes that may occur at different stages of pregnancy – Supportive shared decision‐making	‘These are the risks to the baby … and these are the risks to the heart condition of the pregnancy … once you become pregnant we'll totally support you…’ (S07).
Tailored care (Condition‐focused counselling)	Optimising MLTC management	– Pre‐pregnancy MLTC disease control and health optimisation – Early referral to specialist services – Involvement of multidisciplinary teams	‘When you're thinking about starting a family get into contact, we want to optimise your condition before you become pregnant to ease the pregnancy’ (S05).
Tailored care (Medication planning)	Proactive medication review and planning	– Desire to start a family discussed when initiating or reviewing treatments – Individualised benefit and risk communication – Monitored dose adjustments or switching to pregnancy‐compatible medications – Coordinated multi‐disciplinary team input to support decisions	– ‘It's reassuring them that we need to keep their disease as quiescent as possible, and that there are actually a lot of safe medications’ (S17). – ‘I was on Methotrexate … they were basically like, “You'd have to get an abortion.”’ (W12).
Sensitive and realistic care and support	Mental health support	– Early screening – Timely referral to perinatal mental health services – Integration of mental and physical health	– ‘I had my third [miscarriage] … I had a big breakdown … the GP aid, “Okay, maybe you should try some antidepressants.”’ (W41). – ‘It will be a huge challenge to your mental health … offering perinatal mental review … is something that's totally overlooked’ (S07).
Sensitive and realistic care and support	Non‐medical therapies	– Input from pain management, physiotherapy, and occupational therapy teams – Holistic preparation and comfort	‘I take morphine on a daily basis … the pain team were really amazing … they were really reassuring’ (W17).
Optimising antenatal care	Continuity and coordination of care	– Support transition into pregnancy – Coordination across teams	‘He [GP] made the referral for me and then Obstetrics declined the referral and said there was nothing they could do to help me until I got a positive test … which, to me, is just bonkers’ (W27).
Awareness, knowledge and education	Communication and shared decision‐making	– Respectful, clear dialogue – Recognise women's own knowledge and experiences – Signposting to research and pregnancy registers – Use of evidence‐based resources – Avoid placing responsibility on women to educate professionals about their conditionSupport for shared choices	– ‘It was not nice the whole thing, I was not supported at all … it was like well do it or don't … I didn't know … you're throwing this diagnosis at me, telling me to make a decision that is going to change my life’ (W16). – ‘I'd done so much research … I was able to give the names of all these drugs … I'm on a drug at the moment called Xonvea, which no GP has even heard of’ (W27).
Responsibility	Taking ownership of pregnancy planning	– Proactive pregnancy planning and contraception access during health condition reviews – Pregnancy planning should be considered as part of prescribing practices – Shared accountability across specialities	‘The number of rheumatologists I talk to, I'm like, “Contraception is your business” … the drug regulators made it very clear, it's the prescriber's responsibility’ (S38).
Challenges to care delivery	Addressing lifestyle factors	– Provide woman‐centred, holistic advice tailored to individual circumstances, recognising lifestyle in context and avoiding stigma or blame – Support condition optimisation and pregnancy intention – Offer referrals to specialist services (e.g., dietitian, physio, smoking cessation)	‘It's a much more detailed conversation … where all the questions could be addressed … not just the medical stuff, their general concerns … in a safe and non‐time pressured environment’ (S22).
Enablers of good practice			
Enablers of good practice (Cross‐cutting features)	Communication style	– Respectful, clear and honest (even when there is uncertainty) – Encourages questions and clarifications to support understanding and manage expectations – Enables shared decision‐making – Recognises the role partners, family, or carers can play in discussions and decisions	– ‘…you're throwing this diagnosis at me, telling me to make a decision that is going to change my life, and could make me more poorly’ (W16). – ‘often they're with someone … and they might have some questions’ (S22).
Enablers of good practice (Cross‐cutting features)	Environment	– Ensure privacy and dignity for sensitive discussions, and use non‐maternity settings where possible – Offer flexibility in how care is delivered (e.g., remote/online options) – Create space for support networks (e.g., partner, carer) to be included	‘The setting for that is quite important … ideally you would make it in a non‐maternity setting, but with maternity professionals there…’ (S24).
Enablers of good practice (Cross‐cutting features)	Optimal timing	– Flexible care responsive to pregnancy planning, pregnancy, or change of status	‘It actually takes a long time … that would be good if we met them pre‐pregnancy … women can have time to digest it and consider it, rather than we see them at eight weeks … and the clock is ticking’ (S45).
Enablers of good practice (Cross‐cutting features)	Multidisciplinary coordination	– Clear pathways and communication across primary, secondary, and specialist care – Named professionals or clear points of contact – Teams adapt to changing health needs – Timely referrals	‘He [GP] made the referral … and then Obstetrics declined the referral … it would have been so much better … I tried but it was just declined’ (W27).

Building on our findings, research is needed to co‐develop, implement and evaluate multidisciplinary, integrated care pathways that are both realistic to health professionals and meet the needs of women with MLTC. Such research should identify and address intersectional barriers to accessing care and explore models of care delivery that reduce health inequalities. Additionally, ongoing evaluation is needed to determine the impact of enhanced training for health professionals and adequately funded care pathways on the provision of preconception care, pregnancy outcomes and women's satisfaction with the care and support they receive. We also recognise the importance of understanding preconception care needs from the perspective of male reproductive partners. As part of our original primary study, we conducted a small number of interviews with male partners to explore issues related to preconception care. Findings from these interviews will be reported separately. Further research is urgently needed to inform tailored preconception health education, care and support for male reproductive partners [29].

## 5 Conclusion

This study provides valuable insights into the preconception care experiences of women with MLTC and their healthcare providers and highlights gaps in the availability and quality of care provision for this high‐risk population. Findings illustrate the importance of sensitive and holistic support that includes shared decision‐making, empathy for women and early relationship‐building to enable timely and tailored antenatal care. Some women conduct their own research when preparing for pregnancy due to a lack of comprehensive and consistent advice, which leaves them feeling isolated and unsupported. There is an urgent need to improve the integration of preconception and interpregnancy care into existing services to ensure consistent, high‐quality care for all women with MLTC. All professionals involved in the care of girls and women with MLTC, from paediatrics through the transition to adult services, should share responsibility for delivering preconception care. Public health efforts to raise awareness of preconception health are also essential. The findings underpin the wider MuM‐PreDiCT consortium's work to co‐produce and test a care bundle aimed at improving experiences and outcomes for women with MLTC, their babies and care providers.

## Author Contributions

S.J.H. drafted the manuscript and contributed to the methodology and conceptualisation of the study. D.S., M.S., N.M., S.I.L., S.M., N.M.C.L.M. and Z.W. reviewed and edited the manuscript. N.M. and R.P. provided PPIE input and contributed to reviewing and editing the manuscript. M.S. also provided project administration. K.N., L.L., M.B. and B.T. provided supervision and guidance.

## Ethics Statement

Wales Research Ethics Committee 7 granted National Health Service (NHS) Health Research Authority (HRA) ethical approval.

## Conflicts of Interest

The authors declare no conflicts of interest.

## Data Availability

The authors have nothing to report.
